# Risk of CVD Following Radiotherapy for Head and Neck Cancer: An Updated Systematic Review and Meta-Analysis

**DOI:** 10.3389/fonc.2022.820808

**Published:** 2022-06-01

**Authors:** Ping-Yi Lin, Ping-Chia Cheng, Wan-Lun Hsu, Wu-Chia Lo, Chen-Hsi Hsieh, Pei-Wei Shueng, Li-Jen Liao

**Affiliations:** ^1^ Oral Maxillofacial Surgery, Far Eastern Memorial Hospital, Taipei, Taiwan; ^2^ Head and Neck Cancer Surveillance and Research Group, Far Eastern Memorial Hospital, New Taipei, Taiwan; ^3^ Otolaryngology, Far Eastern Memorial Hospital, Taipei, Taiwan; ^4^ Genomics Research Center, Academia Sinica, Taipei, Taiwan; ^5^ Division of Radiation Oncology, Department of Radiology, Far Eastern Memorial Hospital, New Taipei City, Taiwan; ^6^ Faculty of Medicine, School of Medicine, National Yang-Ming University, Taipei, Taiwan; ^7^ Institute of Traditional Medicine, School of Medicine, National Yang-Ming University, Taipei, Taiwan; ^8^ Department of Electrical Engineering, Yuan Ze University, Taoyuan, Taiwan

**Keywords:** cerebrovascular disease, head and neck cancer, radiotherapy, radiotherapy - adverse effects, systematic review and meta-analysis

## Abstract

**Background:**

The relative risk for cerebrovascular disease (CVD) is increased in patients with head and neck cancer (HNC) treated with radiotherapy (RT). However, the current relative risk for CVD following RT has not been well clarified. The purpose of this study was to analyze the effect of RT and update the risk of CVD following RT in HNC patients through a systematic review and meta-analysis.

**Material and Methods:**

We conducted an online database search and systematic review of observational studies that reported on CVD and extracranial carotid stenosis in patients with HNC who had undergone RT. Articles published in Medline and PubMed from 1980 to 2021 were identified and collected.

**Results:**

Of the forty-seven articles identified from PubMed and forty-four articles identified from 3 systematic reviews, twenty-two studies were included. We found that neck RT was a significant risk factor for CVD (HR 3.97, 95% CI: 2.89-5.45). Patients with HNC treated by RT had an increased OR (7.36, 95% CI: 4.13-13.11) for CVD, and approximately 26% (95% CI: 22%-31%) of HNC patients treated with RT were at risk for CVD with more than 50% reduction in carotid diameter.

**Conclusion:**

The risk of CVD is increased in patients with HNC treated by RT, and recent improvements in RT techniques may have contributed to the decreased risk of CVD. These results suggest that regular follow-up and appropriate screening for CVD should be required for patients with HNC.

## Background

In the United States, cancer-related mortality has declined with improved treatment, and consequently, the number of cancer survivors increased to 17 million in 2019 (1). Due to the increasing number of head and neck cancer survivors, cancer-therapy-related cardiovascular complications impact both morbidity and mortality (2). Among these complications, radiation-induced cerebrovascular disease (CVD) is one of the most important issues.

Radiotherapy (RT) or concurrent with chemoradiation therapy (CCRT) is an essential therapeutic modality for patients with head and neck cancer (HNC). However, CVD in patients with HNC is under-identified and undertreated (3). The increased risk in ischemic CVD following RT has been reported in several cohort studies (4–8). Although previous systematic reviews have been reported, the quantitative method has not been updated, and there are limitations in the study design. Because of the risk of RT-related CVD, we organized a task force to conduct a comprehensive review on the risk of RT-related CVD in HNC survivors.

In the current study, a quantitative meta-analysis of the risk of CVD in post-RT/ CCRT HNC patients was designed and studied. Moreover, the assessment/screening for CVD in post-RT/CCRT HNC patients and the prevention/treatment of CVD in post-RT/CCRT HNC patients were investigated to provide potential clinical applications.

## Material and Methods

**Figure 1:**
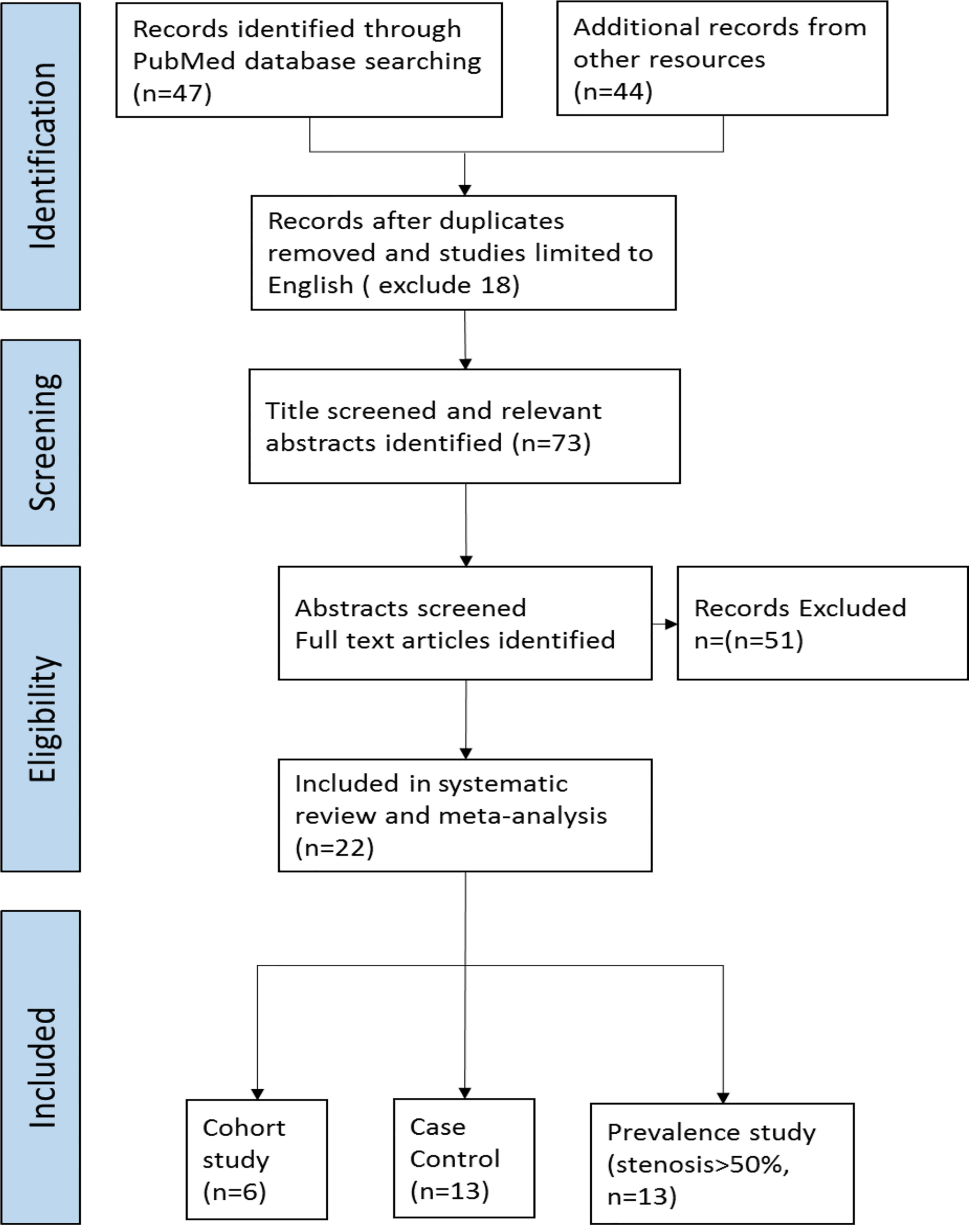
PRISMA flow diagram of searching process.

We conducted a search on Medline and PubMed with the MeSH terms “Cerebrovascular disease AND head and neck cancer AND Radiotherapy (((head and neck cancer) AND radiotherapy [MeSH Terms]) AND Cerebrovascular disease [MeSH Terms] in the PubMed database)” in October 2021 following the PRISMA (Preferred Reporting Items for Systematic reviews and Meta-Analyses) guidelines (**Figure 1**) to identify relevant studies in the published literature. The search was performed for articles published from 1980 to 2021. Additional records from other review articles were also extracted (9–11).

### Literature Inclusion Criteria

1) Studies that were original research; 2) studies that evaluated patients with histopathologically proven head and neck cancer who underwent radiotherapy; 3) studies that provided data about cerebrovascular events, such as carotid stenosis, carotid intima-media thickness or ischemia stroke; 4) studies published between 1980 and 2021; an 5) studies published in English.

### Literature Exclusion Criteria

1) Studies that did not meet our inclusion criteria, 2) studies for which the data had already been published or were duplicate data and 3) studies with incomplete raw data.

### Extracted Information, Excel Spreadsheet, and Information Retrieval

1) The general information extracted included the title, first author, and publication date. 2) The relative risk (RR) or hazard ratio (HR) and 95% CI were extracted for cohort studies; the number of patients with RT-related treatment and the number of patients in the control group were extracted for case–control studies; and the number of cases of CVD among the total number of RT patients was extracted for prevalence studies.

### Statistical Synthesis and Analysis

The hazard ratio (HR) with 95% confidence interval (CI) was calculated to evaluate the risk of CVD in the general population and in those receiving different treatment modalities by using a random-effects model meta-analysis. The odds ratio (OR) with the corresponding 95% CI was used to compare the clinical characteristics of the post-RT vs. non-RT groups. The cumulative incidence of carotid stenosis and the 95% confidence interval (CI) were computed to estimate the prevalence of CVD (more than 50% of carotid artery diameter stenosis). The I-squared statistic was used to assess heterogeneity. An I-squared greater than 50% indicated significant heterogeneity. A random-effects model was used to pool the effect size of significant heterogeneity. A forest plot was used to graphically display the effect size in each study and the pooled estimates. A p value < 0.05 was considered significant. We performed the meta-analysis with two R software packages: “meta” (12) was used for pooling the hazard ratio and OR, while the package “metaphor” (13) was used for meta-regression to elucidate the possible etiology of heterogeneity.

## Results

### Search Results

The search process is shown in **Figure 1**. The initial literature search yielded 91 potentially relevant records after duplicates were removed, 47 from a PubMed search (N=47) and 44 from 3 other systematic reviews (9–11). After screening the titles and abstracts, 73 articles were retrieved for full-text evaluation. Twenty-two studies met the predetermined eligibility criteria and were included in the meta-analysis, as shown in the PRISMA flow diagram.

Twenty-two studies were included (4–7, 14–31). Within the 22 studies, there were six cohort studies, of which two studies reported RR (6, 14), and another four studies reported HR (4, 5, 15, 16). Moreover, there were 13 studies with case–control study designs (7, 17–28), and another three studies (29–31) reported the number of patients with carotid stenosis after neck radiation. A total of 35,160 patients had a history of head and neck cancer treated with radiation therapy. Most patients were diagnosed with laryngeal carcinoma (32%), followed by undesignated head and neck squamous cell carcinoma (18%), oral cancer (17%), nasopharyngeal cancer (14%), oropharyngeal cancer (12%), hypopharyngeal cancer (3%), salivary gland cancer (3%), and nasal cavity or sinus cancer (1%). The imaging modalities used for the detection of carotid stenosis were Doppler ultrasound (most of the included studies) and magnetic resonance angiography (one study) (28). In the cohort study reporting the RR of CVD following radiation, Dorresteijn et al. (2002) (6) reported that radiation to the neck significantly increased the RR (5.6, 95% CI: 3.1-9.4) of stroke compared to the general population. Haynes et al. (2002) (14) also reported that radiation to the neck with/without surgery increased the relative risk of stroke (RR 2.09, 95% CI: 1.28-3.22) compared to that of the general population (**Table 1** **).**

**Table 1:** Summary of the 22 included studies.

	Author	Treat1	Treat2/Control	RR or HR	lower.HR	upper.HR	Country	Cancer	Remark	Study type	Methods for CVD	Treat1 incidence (%)	Treat2 incidence (%)	RT dose (Gy)
1	Haynes (2002) (14)	RT+-SUG	Population	RR 2.09	1.28	3.22	USA	HNC	Stroke	Retro	No. of stroke	4.8	Nli	64
2	Dorresteijn (2002) (6)	RT	Population	RR 5.6	3.1	9.4	Netherland	HNC	Stroke	Retro	No. of stroke	3.8	Nil	50-66
3	Smith (2008) (4)	RT	Surgery	HR 1.50	1.18	1.90	USA	HNC	CVD	Retro	No. of stroke, carotid revascularization, or stroke death	4	3	Nil
		RT	Surgery + RT	HR 1.42	1.14	1.77	USA	HNC		Retro		4	3	Nil
4	Arthurs (2016) (5)	RT	Surgery	HR 1.70	1.41	2.05	Canada	HNC	Stroke	Retro	No. of stroke	Nil	Nil	Nil
5	Chen (2019) (15)	RT	Population	HR3.97	2.89	5.44	Taiwan	NPC	Stroke	Retro	No. of stroke	Nil	1.3	Nil
		CCRT	Population	HR 3.26	2.43	4.38	Taiwan	NPC		Retro		Nil	1.3	Nil
6	Swisher (2019) (16)	RT	Surgery	HR 1.75	1.04	2.96	USA	Glottic cancer	Fatal CVA	Retro	No. of death from CVA	2.8	1.5	Nil
	Author	Case/RT	Noncase/RT	Case/Control	Noncase/Control		Country	Cancer	Grade of carotid stenosis	Study type	Methods for CVD			RT dose (Gy)
7	Moritz (1990) (17)	16	37	2	36		USA	HNC	50%	Retro	Doppler US			>50
8	Cheng (2000) (18)	35	61	8	88		HK	NPC	70%	Retro	Doppler US			64-72
9	Carmody (1999) (19)	5	18	2	44		USA	HNC	70%	Retro	Doppler US			Nil
10	Lam_H&N (2001) (20)	24	56	0	58		HK	NPC	50%	Retro	Doppler US			56.6
11	Lam_Cancer (2001) (21)	21	50	0	51		HK	NPC	50%	Retro	Doppler US			Nil
12	Chang (2009) (22)	38	154	0	98		TW	HNC	50%	Retro	Doppler US			>60
13	Greco (2012) (23)	9	30	3	51		Italy	HNC	50%	Pros	Doppler US			Nil
14	Dubec (1998) (24)	17	28	13	335		Canada	HNC	50%	Retro	Doppler US			59.5
15	Cheng (2004) (25)	43	87	22	73		HK	HNC	50%	Retro	Doppler US			60
16	Martin (2005) (26)	6	34	1	39		Canada	HNC	60%	Retro	Doppler US			>35
17	Brown (2005) (7)	8	36	3	41		USA	HNC	50%	Pros	Doppler US			>45
18	Tai (2013) (27)	8	39	1	46		Malaysia	NPC	50%	Retro	Doppler US			66
19	Zhou (2015) (28)	33	111	2	98		China	NPC	50%	Pros	MR angiography			66
20	Griewing (1995) (29)	4	12	NA	NA		Germany	HNC	50%	Retro	Doppler US			56.2
21	Steele (2004) (30)	16	24	NA	NA		USA	HNC	50%	Pros	Doppler US			64.2
22	Carpenter (2018) (31)	58	308	NA	NA		USA	HNC	50%	Retro	Doppler US			48

HNC (head and neck cancer), NPC (nasopharyngeal carcinoma), HK (Hong Kong), TW (Taiwan), USA (United States of America), Retro (retrospective study), Pros (prospective study), No. of stroke (Numbers of stroke), Pop (population), RT (radiotherapy), Surg (surgery).

**Figure 2:**
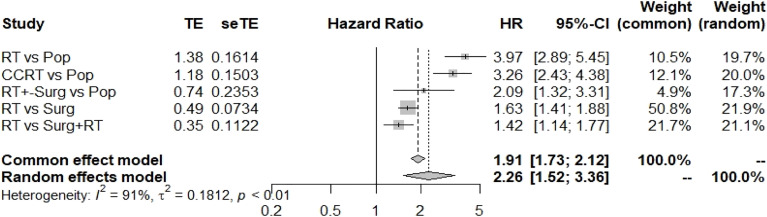
Summary of the hazard ratios for CVD for different treatment methods. Pop, population; RT, radiotherapy; Surg, surgery.

Comparing the HR of CVD in the general population (Pop) with that of patients receiving different treatment modalities, RT alone for head and neck patients indeed increased the risk of CVD [HR 3.97(2.89-5.45)] compared with that in the general population in the random-effects model (**Figure 2** **)**. Additionally, concurrent chemoradiation therapy also increased the HR [3.26 (2.43-4.38)] for CVD. Interestingly, compared to RT with surgery, RT alone significantly increased the risk of CVD (HR: 1.42, 1.14-1.77) (**Figure 2** **)**.

**Figure 3:**
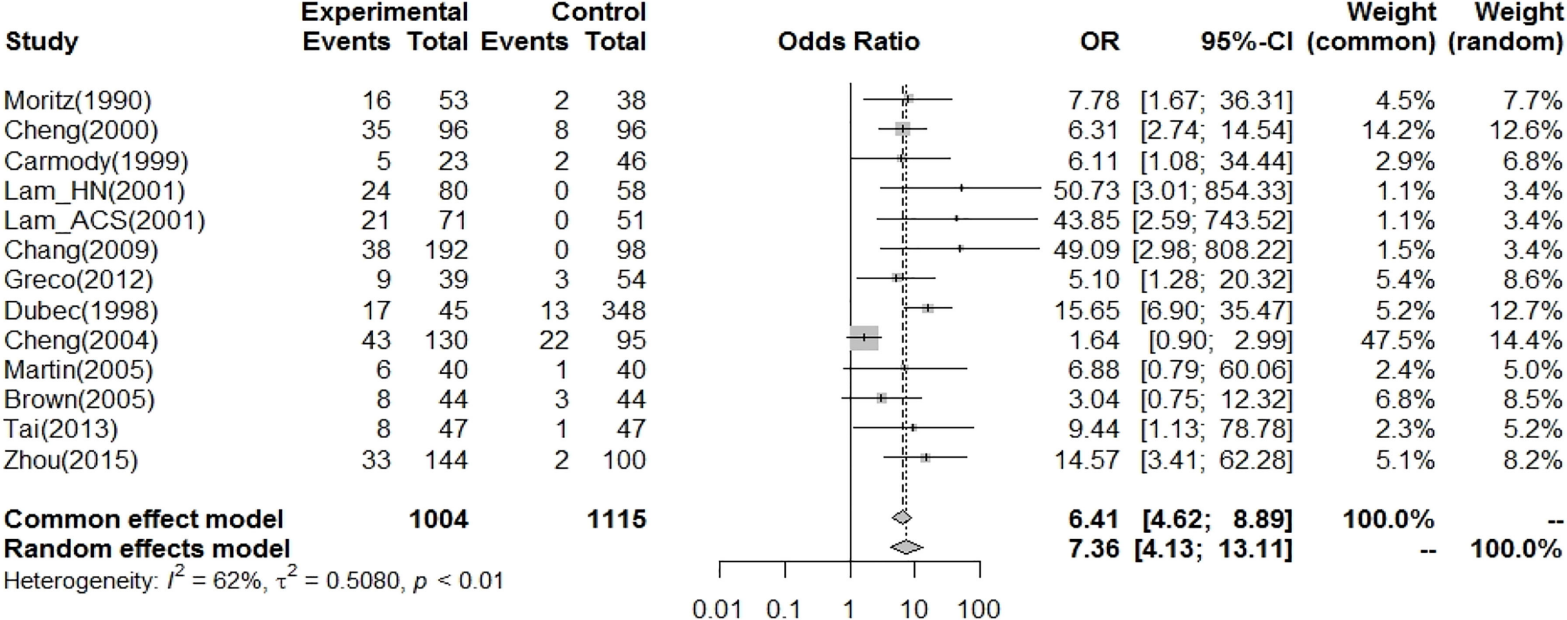
In case–control studies, the pooled OR for radiation-related CA vasculopathy (carotid artery stenosis>50%~%70 as risk) was 7.36 (95% CI: 4.13-13.11).

Thirteen case–control studies reported carotid stenosis in patients with HNC (**Table 1**). The RT-related CCA vasculopathy results are shown in **Figure 3**. The pooled OR (odds ratio) for an increased risk of CVD was 7.36 (4.13-13.11) using a cutoff point of 50% carotid artery stenosis in the random-effects model. However, there was significant heterogeneity among studies.

**Figure 4:**
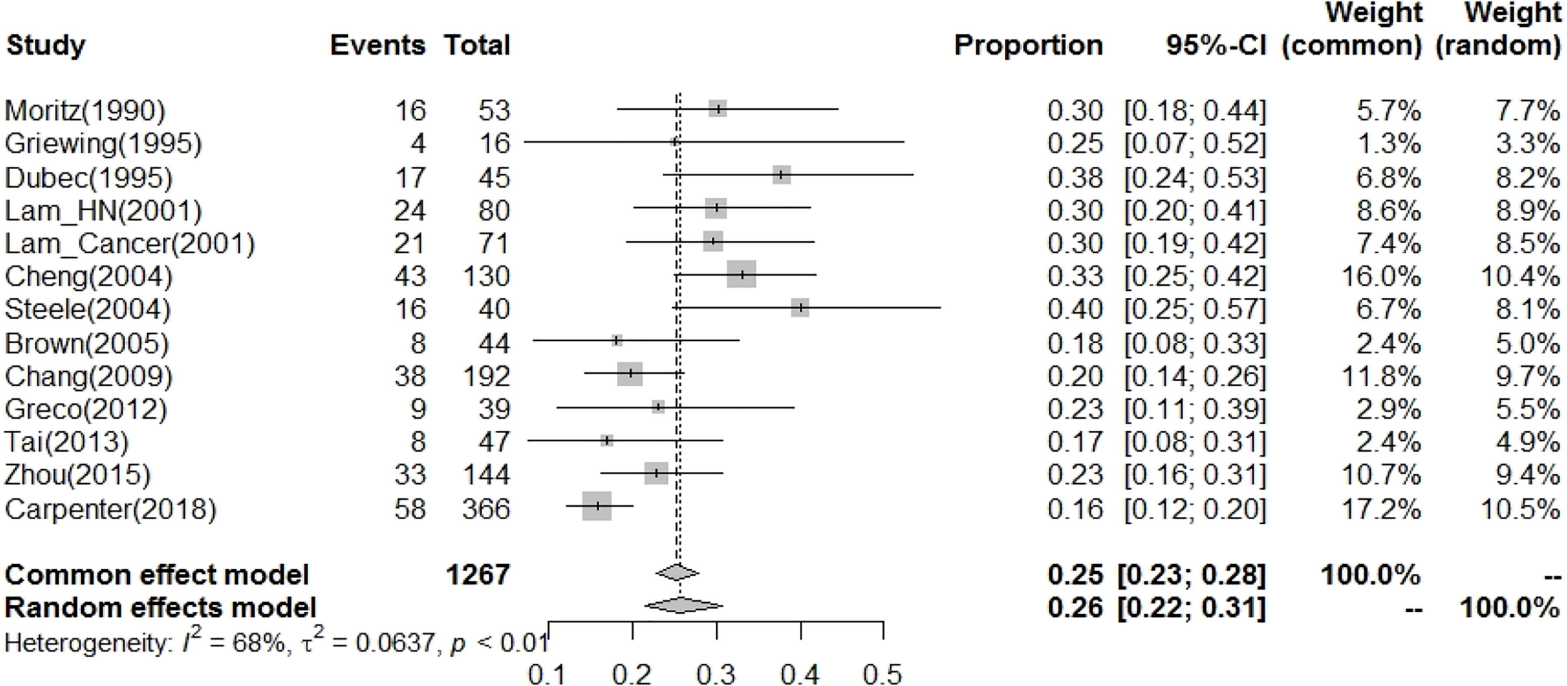
The prevalence of CVD risk (CA stenosis>50% as increasing risk for CVD) for patients after radiotherapy to the neck was 26% (95% CI: 22%-31%).

The current study demonstrated that the prevalence of CVD with more than 50% carotid stenosis in post-RT HNC patients was 26% (95% CI: 22%-31%, **Table 2** and **Figure 4** **)**. In meta-regression analysis to clarify the possible factors contributing to the heterogeneity among studies, we found that the publication year was a significant factor that contributed to the heterogeneity (p-value < 0.001, **Table 2**). In studies published before 2004, the prevalence of CVD with more than 50% carotid stenosis in post-RT HNC patients was 33% (95% CI: 29%-38%).

**Table 2:** Results of meta-regression analysis with the R package metafor, showing that the year of publication and subsites of cancer were significant contributing factors to the heterogeneity.

Characteristics	% of CA stenosis>50%	z-val	p-val
**Publication year**		5.0234	<.0001
Before 2004	33% (29-38%)		
After 2004	19% (16-22%)		
**Overall**	26% (22-31%)		

CA, carotid artery.

## Discussion

We collected multiple studies and combined different study designs to clarify the effects of radiation effect to the neck. We concluded that radiation is a significant risk factor for CVD (HR 3.97, 95% CI: 2.89-5.45). Post-RT head and neck cancer patients had an increased OR (7.36, 95% CI: 4.13-13.11) for the risk of CVD, and approximately 26% of patients were at risk for CVD, defined as having more than 50% carotid diameter reduction. Our findings provide scientific evidence and are helpful for the development of protocols for the diagnosis and prevention of CVD.

Another meta-analysis of eight studies reported the pooled relative risk (9) and RR (7.54, 95% CI: 3.65-15.59) for high-grade carotid stenosis. Because the total number of patients at risk was not followed prospectively, the effect size should be calculated as the OR (32). Our OR for carotid stenosis more than 50% results is similar (7.36, 95% CI: 4.13-13.11).

The cost-effectiveness of carotid artery stenosis screening depends on the prevalence. One study revealed that the prevalence of carotid stenosis in the general population was 0% to 7.5% for moderate stenosis (carotid stenosis >50%) and 0% to 3.1% for severe stenosis (carotid stenosis >70%) (33). CVD screening is recommended if the prevalence of carotid artery stenosis is more than 20% (34). Previous meta-analysis reported that the prevalence of carotid stenosis in post-RT HNC patients was 25% (95% CI: 19%-32%) for moderate stenosis, 12% (95% CI: 7%-17%) for severe stenosis, and 4% (95% CI: 2%-8%) (11) for carotid occlusion. In our study, we estimated that the pooled prevalence for carotid stenosis (>50% luminal stenosis) was 26% (95% CI: 22%-31%). This result indicates that screening in post-RT HNC patients is necessary.

CVD is an underestimated condition for head and neck cancer patients (3). Okoye et al. reported that approximately 23% (27/115) of head and neck cancer patients have cardiovascular disease at diagnosis. Among these patients, 15% (17/115) had coronary artery disease and 9% (10/115) had carotid artery disease (35). A high prevalence of cardiovascular disease risk factors at HNC diagnosis requires personalized lifestyle changes and risk factor modifications to achieve LDL, blood pressure and blood sugar targets as early as possible (36).

Radiation-related carotid vasculopathy is a dynamic and progressive process that can result in the depletion of parenchymal and vascular endothelial cells, with both macro- and microvascular effects (37). Oxidative stress caused by reactive oxygen species promotes endothelial dysfunction and inflammatory changes in the radiation field (38). Accordingly, RT induces the release of thromboxane (39) and increases the level of von Willebrand factor, which causes platelet adhesion to endothelial cells and predisposes patients to arterial thrombosis (40). Simonetto et al. reported an increase in carotid intima media thickness (CIMT) one year after radiation for hypopharyngeal cancers (41). Therefore, it is necessary to screen the carotid artery one year after neck radiation. The late effects of radiation to the carotid artery will progress (42); therefore, regular extracranial color-coded duplex sonography examination is reasonable.

Neck irritation will induce inflammation in the arteries; however, the mechanism through which this occurs is still poorly understood. To date, there are no guidelines for medication in the prevention of radiation-associated CVD. In radiotherapy-induced carotid artery vasculopathy, CIMT was reported to be related to LDL cholesterol levels (43). According to a retrospective study, statin use was associated with a significant reduction in the incidence of stroke of 32% among cancer patients after radiation to the thorax, head and neck (44). There is growing evidence of anti-inflammatory medication to prevent radiation-associated CVDs, such as statins, colchicine and aspirin (45). More evidence is necessary for anti-inflammatory medication to prevent radiation-associated CVD.

The treatment of head and neck cancer requires a multidisciplinary team, including head and neck surgeons, radiation oncologists, hemato-oncologists and cardio-oncologists. Novel models for comprehensive head and neck cancer survival are necessary to provide a multidisciplinary approach to the prevention, screening and treatment of radiation-related CVD.

Due to technical innovations, the prevalence of radiation-related carotid vasculopathy may be decreased. In our studies, we found that publication year was an important factor in the heterogeneity among studies. One study reported that IMRT can reduce the risk for CVD compared to 2D RT (46). However, Addison et al. reported that patients with HPV-related head and neck cancer who underwent radiation had an increased risk for CVD (HR 4.4, 95% CI: 1.5-13.2) compared to HPV-negative patients (47). In addition, advances in head and neck cancer treatment have led to increased survival. Radiation-related CVD will still be an important issue in the future due to the emergence of HPV-related HNC.

There are limitations in the current study. First, there was significant heterogeneity among the collected studies, which may be due to various radiation dosages, radiation protocols, radiation techniques, and follow-up times. However, there were no sufficient information about the radiation dosages, protocols, and techniques from the included studies. The follow-up duration of the included studies was varying. The radiation dosages were either recorded as main tumor, neck or carotid region. The radiation protocols and techniques were mostly not mentioned. Thus, we cannot achieve further analysis. Second, the enrolled studies were nonrandomized and were observation studies. Only four of the included reports were prospective cohort studies, and others were retrospective studies. Third, there are still no solid guidelines for screening and treatment, and further studies are necessary to develop cost-effective methods in the management of radiation-related CVD. Fourth, the timeframe of the included articles is very large, the CVD risk may be change by radiation technique, HPV status and patients’ survival condition.

## Conclusion

The included studies demonstrated that the prevalence of CVD with more than 50% carotid stenosis in post-RT HNC patients was 26%. Based on our analysis, RT for HNC patients can increase the risk of CVD. To combat this complication, close follow-up studies and appropriate screenings for CVD are recommended for HNC patients who receive RT

## Data Availability Statement

The raw data supporting the conclusions of this article will be made available by the authors, without undue reservation.

## Author Contributions

1. Conceived and designed the study: P-CC, W-LH, and L-JL; 2. Collected the data: P-YL, P-CC, W-LH, W-CL, C-HH, P-WS, and L-JL; 3. Performed the analysis P-CC, W-LH, and L-JL; 4. Wrote the paper: P-YL, P-CC, W-LH, W-CL, C-HH, P-WS, and L-JL. All authors contributed to the article and approved the submitted version.

## Funding

This work was supported by grants from the Far Eastern Memorial Hospital (FEMH -2021-C-011, PI20190002) and National Science Council of the Republic of China (MOST-109-2314-B-418-004).

## Conflict of Interest

The authors declare that the research was conducted in the absence of any commercial or financial relationships that could be construed as a potential conflict of interest.

## Publisher’s Note

All claims expressed in this article are solely those of the authors and do not necessarily represent those of their affiliated organizations, or those of the publisher, the editors and the reviewers. Any product that may be evaluated in this article, or claim that may be made by its manufacturer, is not guaranteed or endorsed by the publisher.

## Supplementary Material

Supplementary Figure 1 The prevalence of CVD risk (CA stenosis>50% as increasing risk for CVD) for patients after radiotherapy to the neck was 33% (95% CI: 29%-38%) among studies published before 2004.

Supplementary Figure 2 The prevalence of CVD risk (CA stenosis>50% as increasing risk for CVD) for patients after radiotherapy to the neck was 19% (95% CI: 16%-22%) among studies published after 2004.
