# Supplementary figures and images for: Risk of CVD Following Radiotherapy for Head and Neck Cancer: An Updated Systematic Review and Meta-Analysis
Source: Front Oncol. 2022 Jun 1;12:820808. doi: 10.3389/fonc.2022.820808 (PMC9198239; doi:10.3389/fonc.2022.820808)

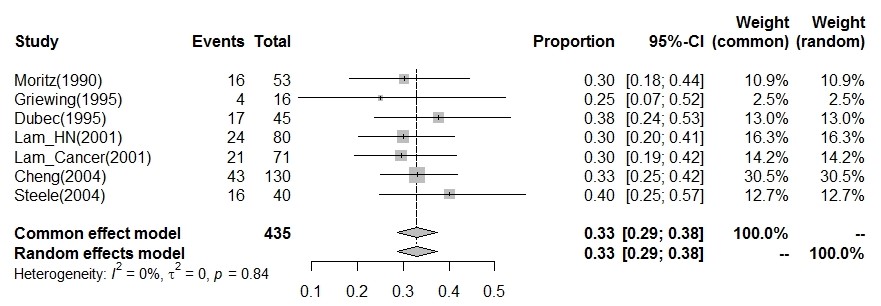

Supplement: Supplementary Figure 1 — The prevalence of CVD risk (CA stenosis>50% as increasing risk for CVD) for patients after radiotherapy to the neck was 33% (95% CI: 29%-38%) among studies published before 2004. [file Image_1.jpg]

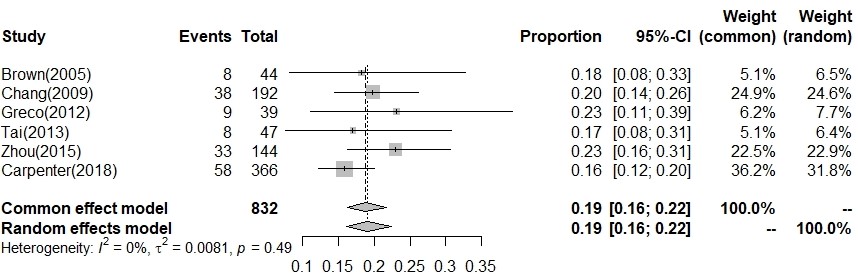

Supplement: Supplementary Figure 2 — The prevalence of CVD risk (CA stenosis>50% as increasing risk for CVD) for patients after radiotherapy to the neck was 19% (95% CI: 16%-22%) among studies published after 2004. [file Image_2.jpg]
